# Theory, methods, and operational results of the Young Women’s Health History Study: a study of young-onset breast cancer incidence in Black and White women

**DOI:** 10.1007/s10552-021-01461-x

**Published:** 2021-07-22

**Authors:** Ellen M. Velie, Lydia R. Marcus, Dorothy R. Pathak, Ann S. Hamilton, Ralph DiGaetano, Ron Klinger, Bibi Gollapudi, Richard Houang, Nicole Carnegie, L. Karl Olson, Amani Allen, Zhenzhen Zhang, Denise Modjesk, Gwendolyn Norman, Darek R. Lucas, Sapna Gupta, Hallgeir Rui, Kendra Schwartz

**Affiliations:** 1 Zilber School of Public Health, University of WI – Milwaukee, 1240 N. 10th Street, Milwaukee, WI 53201 USA; 2 Departments of Medicine and Pathology, Medical College of Wisconsin, 9200 W. Wisconsin Ave, Milwaukee, WI 53226 USA; 3 Department of Epidemiology and Biostatistics, College of Human Medicine, Michigan State University, 909 Wilson Road Room B601, East Lansing, MI 48824 USA; 4 Department of Preventive Medicine, Keck School of Medicine, University of Southern California, 2001 N. Soto St, Los Angeles, CA 90089-9239 USA; 5 Westat Inc., 1650 Research Blvd, Rockville, MD 20850 USA; 6 Department of Education, Michigan State University, 620 Farm Ln, East Lancing, MI 48824 USA; 7 Department of Mathematics, Montana State University, 732 Grant St, Bozeman, MT 59717 USA; 8 Department of Physiology, Michigan State University, 567 Wilson Rd, East Lansing, MI 48824 USA; 9 Departments of Community Health Sciences and Epidemiology, School of Public Health, University of California Berkeley, 2121 Berkeley Way, Berkeley, CA 94720 USA; 10 Division of Oncological Sciences, Knight Cancer Institute, Oregon Health & Science University, 3181 SW Sam Jackson Park Rd, Mail Code: KCRB-PROS, Portland, OR 97239 USA; 11 College of Liberal Arts and Sciences, Wayne State University, 4841 Cass Avenue, Detroit, MI 48201 USA; 12 Cancer Research Informatics Core, University of Southern California Norris Cancer Center, NRT LG507, 1450 Biggy St, Los Angeles, CA 90033 USA; 13 Department of Pathology, Medical College of Wisconsin, 8701 Watertown Plank RD., Milwaukee, WI 53226 USA; 14 Department of Family Medicine and Public Health Sciences, Wayne State University, 3939 Woodward Ave, Detroit, MI 48201 USA

**Keywords:** Breast cancer, Young-onset breast cancer, Epidemiology, Life-course, Health status disparities, Premenopause

## Abstract

**Purpose:**

The etiology of young-onset breast cancer (BC) is poorly understood, despite its greater likelihood of being hormone receptor-negative with a worse prognosis and persistent racial and socioeconomic inequities. We conducted a population-based case–control study of BC among young Black and White women and here discuss the theory that informed our study, exposures collected, study methods, and operational results.

**Methods:**

Cases were non-Hispanic Black (NHB) and White (NHW) women age 20–49 years with invasive BC in metropolitan Detroit and Los Angeles County SEER registries 2010–2015. Controls were identified through area-based sampling from the U.S. census and frequency matched to cases on study site, race, and age. An eco-social theory of health informed life-course exposures collected from in-person interviews, including socioeconomic, reproductive, and energy balance factors. Measured anthropometry, blood (or saliva), and among cases SEER tumor characteristics and tumor tissue (from a subset of cases) were also collected.

**Results:**

Of 5,309 identified potentially eligible cases, 2,720 sampled participants were screened and 1,812 completed interviews (682 NHB, 1140 NHW; response rate (RR): 60%). Of 24,612 sampled control households 18,612 were rostered, 2,716 participants were sampled and screened, and 1,381 completed interviews (665 NHB, 716 NHW; RR: 53%). Ninety-nine% of participants completed the main interview, 82% provided blood or saliva (75% blood only), and SEER tumor characteristics (including ER, PR and HER2 status) were obtained from 96% of cases.

**Conclusions:**

Results from the successfully established YWHHS should expand our understanding of young-onset BC etiology overall and by tumor type and identify sources of racial and socioeconomic inequities in BC.

**Supplementary Information:**

The online version of this article contains supplementary material available (10.1007/s10552-021-01461-x).

## Introduction

In the United States (US), nearly one quarter of annual breast cancer (BC) cases occur in women under 50 years of age and the incidence is increasing [1, 2]. The etiology of BC varies by age [3, 4] and is poorly understood in young-onset BC [3, 5–8]. Breast tumors are also now recognized to have different histopathologic and molecular characteristics with heterogeneous etiology, prognosis, and treatment [9–12]. Tumors in young women are also more likely to present at a later stage, have a worse prognosis, and be hormone receptor-negative (HR-)[13–15]. Non-Hispanic White (NHW) and non-Hispanic Black (NHB) women have the highest incidences of BC in the U.S. [2] and racial and socioeconomic inequities in BC also persist [16–18].

Racial inequities exist in the U.S. in overall BC mortality and incidence, particularly in younger women, and there are unequal distributions of tumor subtypes. Overall BC mortality was 40% higher in NHB compared to NHW women during 2013–2017 [17] and this inequity is particularly pronounced among women < 50 years of age, where mortality was 82% higher in NHB compared to NHW women in 2018 [19]. Though overall incidence of BC among NHB women has historically been lower than NHW women, rates are now nearly equal [2], and among the youngest women (aged < 40 years) incidence rates have consistently been higher among Black women [2, 20]. Among women < 50 years of age, NHB women also had a 90% higher incidence of the most aggressive HR-/HER2- (i.e., triple-negative (TNBC)) tumors compared to NHW women in 2012–2016 [2]. Studies examining racial residential segregation have observed that among Black women, both a lower [21] and higher [22] proportion of Black residents in census tracts is associated with a higher odds of TNBC. Everyday experiences of discrimination have also been associated with increased incidence of BC among Black women, particularly among those aged < 50 years [23], potentially contributing to an explanation for observed patterns of racial residential segregation and TNBC [22].

Socioeconomic inequities in BC mortality and incidence also exist. Poorer women have historically had lower mortality from BC at all ages [18]; however, mortality from BC has steadily *increased* since 1950 among women residing in disadvantaged census tracts and *decreased* among women in affluent tracts[18]) such that, by 2013, BC mortality in the most disadvantaged tracts was 6% higher than in the most affluent tracts [18]. The incidence of BC overall has also increased among women residing in the most disadvantaged counties more rapidly than among women in the most affluent counties: from 1981–1990 to 2001–2010, incidence increased by 15% in the most disadvantaged and only by 9% in the most affluent counties [24]. Black and White women residing in the most disadvantaged counties (> 20% poverty) also had a higher prevalence of HR- BC relative to women residing in wealthier counties (< 10% poverty) in 2004–2007 [25]; this is most pronounced for NHB compared to NHW women < 50 years old (HR-/HR + ratio = 1.51, 95% Confidence Interval (CI): 1.20, 1.90) [25]. Similar patterns are seen at the census-tract level: women residing in tracts with intermediate and low compared to high socioeconomic status index had 1.81 (95% CI 1.20, 2.71) and 1.95 (95% 1.27–2.99) relative risk ratios for TNBC, respectively, in 2005–2017 [21].

**Fig. 1:**
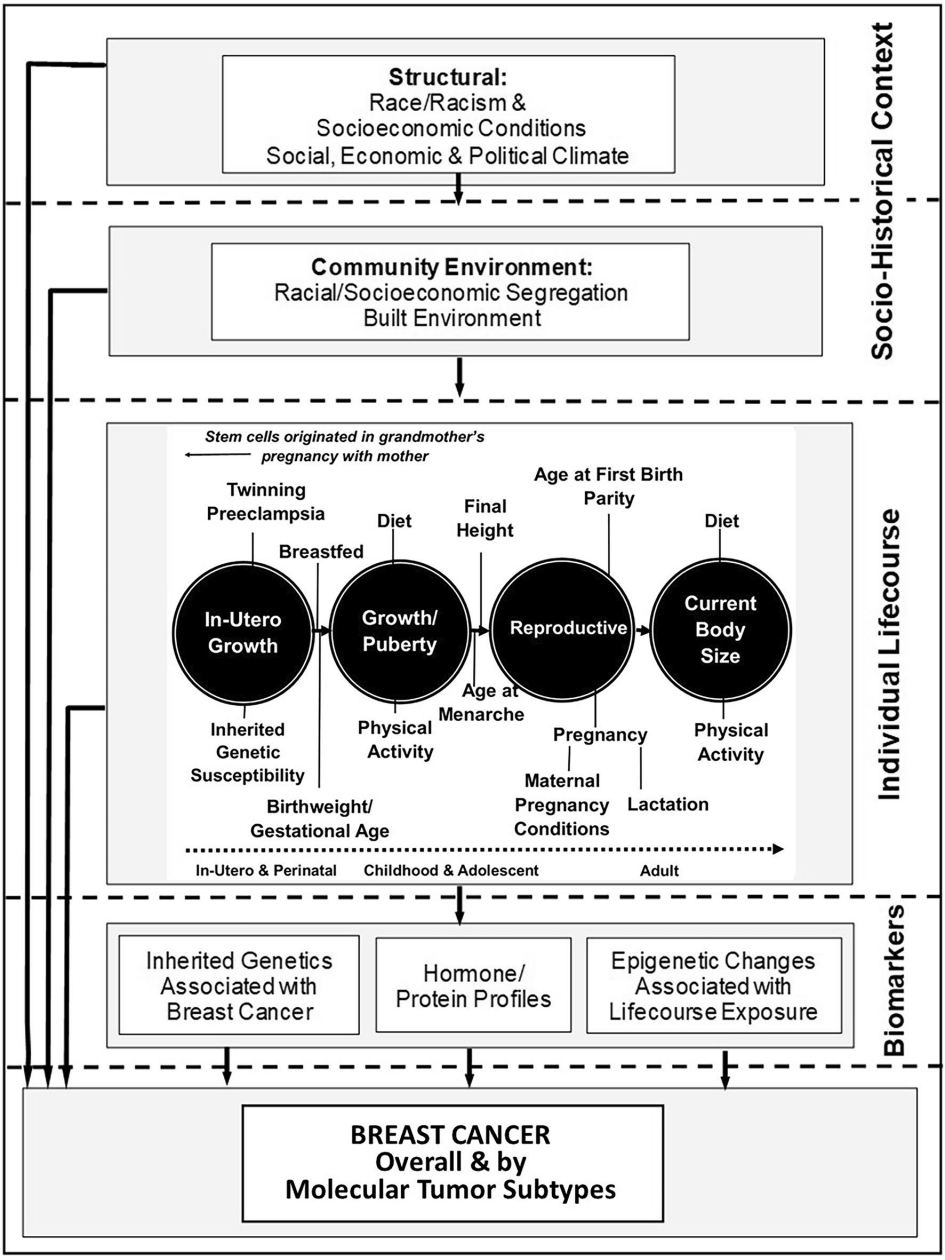
YWHHS conceptual framework: socio-historical context, life-course reproductive and energy balance factors, and breast cancer risk among young non-Hispanic Black and White women

Few modifiable factors have been identified to inform BC prevention strategies [26], particularly in young women [9, 27–31] and by tumor type [9, 13], or to explain racial and socioeconomic inequities in BC incidence [32–34]. We conducted a population-based case–control study of BC risk among NHB and NHW women aged < 50 years old from diverse socioeconomic backgrounds in the US: The Young Women’s Health History Study (YWHHS). Our research is informed by an eco-social theory of health, which situates health outcomes—particularly those between groups—within a complex socio-historical context; eco-social theory seeks to identify the pathways through which that context is embodied [35, 36]. Further, we recognize racism is a potent social determinant that continues to regulate differences in exposures to socioeconomic and other opportunities by race, thereby contributing to racial health inequities in the U.S. [37, 38]. We hypothesize that socio-cultural factors related to race and socioeconomic position determine exposures over the life-course (e.g., reproductive and energy balance factors) that modify biology and, in turn, risk for young-onset BC tumor types (Fig. 1) [22, 36, 38–44]. In this paper, we document details of the YWHHS study design, life-course measures collected, data collection methods, response and cooperation rates, and provide a description of our final study population.

## Methods

### Overall study objectives

The primary objectives of the YWHHS were to provide insight into modifiable early life and life-course factors associated with young-onset (< 50 years) BC risk and to understand racial and socioeconomic inequities in BC risk in the U.S. [40, 44–47]. We are investigating: (1) the association between early life and life-course factors and risk for BC overall and by tumor subtypes among young NHB and NHW women [9, 27–32], (2) the potentially modifying effects of the socio-historic context of race/ethnicity (hereafter “race”) and life-course socioeconomic position (SEP) on BC risk, and have also (3) created a bio-repository of blood (or saliva) and breast tumor tissue for current and future study of the contribution of biomarkers, gene-environment interactions, and gene expression on BC risk in young women.

### Overall study design

BC cases were identified from the metropolitan Detroit (Oakland, Wayne, and Macomb counties) and Los Angeles County Surveillance, Epidemiology and End Results (SEER) registries diagnosed between 2010 and 2015. Controls were identified through area-based sampling from the 2010 Census and matched to cases by study site, age, and race. Primary data collected included: (1) an in-person computer-assisted personal interview (CAPI) conducted with a life history calendar, (2) anthropometric measurements, (3) blood collection (or saliva when not available) and related questionnaire, (4) SEER tumor type information, including ER, PR and HER2 status, and (5) breast tumor tissue collected from participants’ BC surgeries. Additional collected data included: (6) an interviewer-completed built environment survey of participants’ neighborhoods, (7) a survey completed by participants’ primary childhood caregiver, and (8) childhood photos of body size. We also requested (9) permission to obtain information from the health department(s) where women gave birth and (10) where she was born, and (11) most recent mammogram reports from healthcare providers. Participation in the main study questionnaire was necessary for enrollment; all other study components were optional. This study protocol was approved by the Institutional Review Boards at the University of Wisconsin—Milwaukee (UWM); Michigan State University (MSU); Wayne State University (WSU); the Michigan Department of Community Health; University of Southern California (USC); the California Committee for the Protection of Human Subjects (CPHS); and for the Medical College of Wisconsin (MCW), IRB oversight was deferred to UWM. The California Cancer Registry also approved the study.

### Study organization

The YWHHS Coordinating Center (initially hosted at MSU, moved to UWM in 2014) were responsible for study design, development, and oversight of the study tracking system. Westat, a research services corporation, and study collaborators developed the control sampling design, oversaw identification and recruitment of control participants, and created final study sample weights. Final recruitment, in-person interviews, and biospecimen collection were conducted at two field sites: Los Angeles County (at USC) and metropolitan Detroit (at WSU). A community advisory panel was assembled and consulted about data collection materials and study methodologies.

#### Eligibility criteria (see Table 1)

**Table 1:** Eligibility criteria for cases of breast cancer and controls, Young Women’s Health History Study

Cases	Controls
Eligibility criteria:	Eligibility criteria:
1. Identified as female by SEER Registry	1. Identified as female by household roster
2. 20–49 years of age at reference date	2. 20–49 years of age at reference date
3. Race/ethnicity: self-reported Non-Hispanic Black or non-Hispanic White^1^	3. Race/ethnicity: self-reported Non-Hispanic Black or non-Hispanic White^2^
4. Resident of metropolitan Detroit or Los Angeles County at reference date	4. Resident of metropolitan Detroit or Los Angeles County at reference date
5. Born in the U.S	5. Born in the U.S
6. No previous diagnosis of in situ or invasive breast cancer	6. No previous diagnosis of in situ or invasive breast cancer
7. No previous cancer diagnosis except for cervical in situ or common skin cancer	7. No previous cancer diagnosis except for cervical in situ or common skin cancer
8. Not residing in an institution (e.g., prison, shelter, nursing home) at reference date	8. Not residing in an institution (e.g., prison, shelter, nursing home) at reference date
9. Physically and mentally able to complete the interview	9. Physically and mentally able to complete the interview
10. Able to complete interview in English	10. Able to complete interview in English
11. Diagnosed with histologically confirmed invasive BC by SEER between 1 September 2010 and 31 August 2015 (ICD-9-CM code C50.0-C50.9, excluded breast lymphoma, Paget’s disease, mesenchymal tumors, including sarcomas, and hemangiosarcoma's of the breast: 8800–8805, 8540/3, 8541/3, 8542/3, 8543/3, 9000–9805, 9820–9989)	
Reference date:	Reference date:
Date of first microscopic cytologic/histologic BC diagnosis	Four months prior to screening

^1^For cases, race/ethnicity was initially determined by SEER– derived from medical report or hospital admissions. Participants with “Hispanic” or “Arab American” last names based on SEER last name lists [74] at both study sites and participants with “Asian” last names based on SEER lists in LA County were considered ineligible

^2^For controls, race/ethnicity was initially reported on the household roster (potentially by proxy) based on Census 2010 as “Hispanic or Latina origin” and as many races as applied: “Black/African American, White, Asian, Native Hawaiian/other Pacific Islander, American Indian/Alaska Native, or Other [51]. Westat also applied SEER Hispanic surname lists in LA. Final race/ethnicity determination was self-reported on the screener. Participants were asked to report their ethnicity as “Hispanic or Latina origin,” and then to select the race they identified with most: “Black or African American; White; American Indian or Native American or Alaska Native; Arab American or Chaldean; East Asian or Southeast Asian; Asian Indian or South Asian; Native Hawaiian or other Pacific Islander; Some Other Group; Refused; Don’t know.” Participants who did not identify as “Hispanic or Latina origin” and those who identified as “Black or African American” or “White” were considered eligible

NOTE: We use the terms Black and African American interchangeably [75]

#### Study tracking system

A centralized computer system that tracked all corresponding study data and biospecimens was adapted and managed for YWHHS by the USC Cancer Research Informatics Core (CRIC).

#### Ascertainment, sampling, recruitment, and screening

**Fig. 2:**
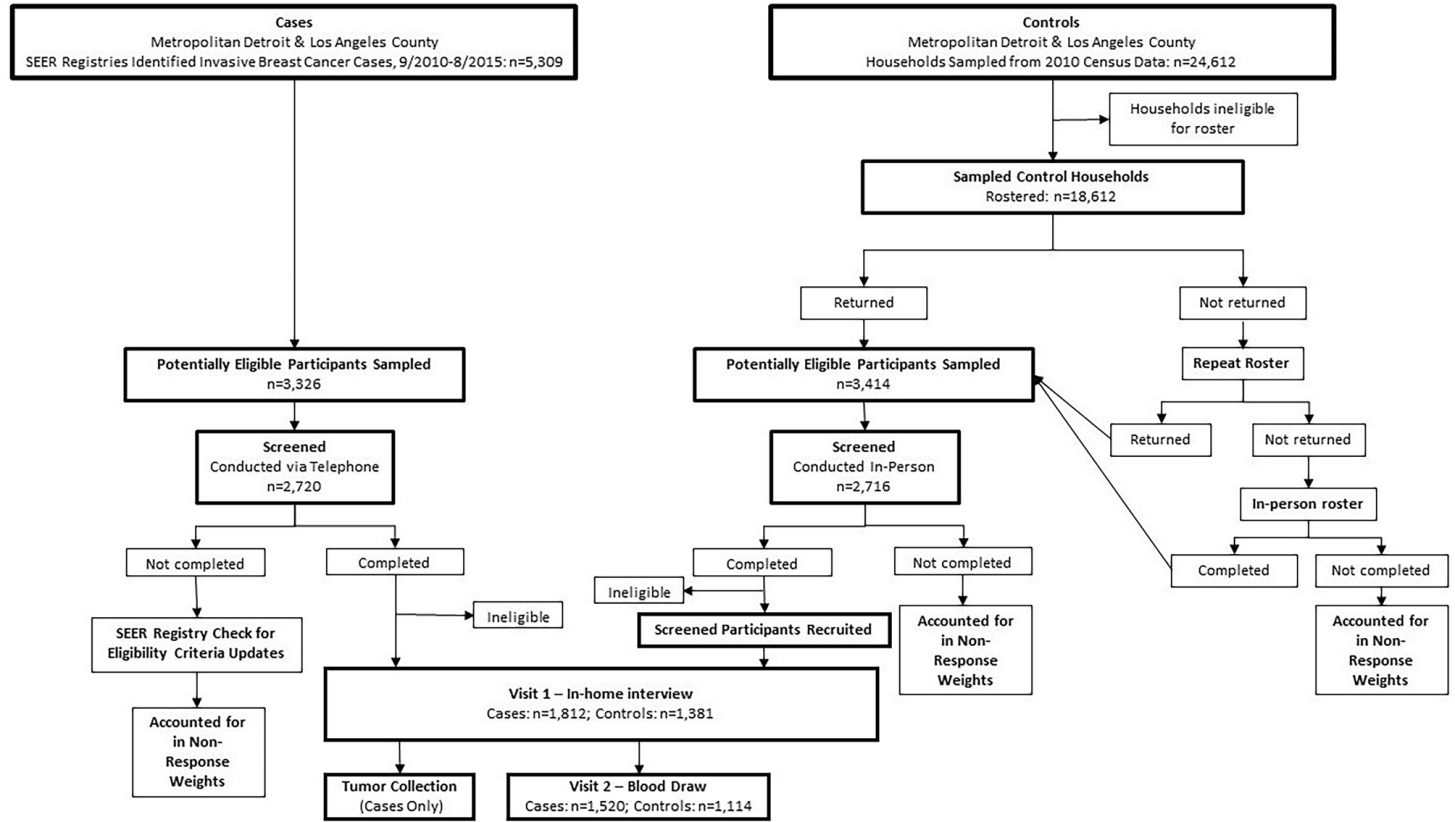
Control and case sampling, eligibility, and recruitment: Young Women’s Health History Study

Ascertainment, sampling, recruitment, and screening activities for cases and controls are outlined in Fig. 2.

#### Cases

Potentially eligible cases were identified by the Metropolitan Detroit Cancer Surveillance System (MDCSS) SEER registry and the LA County Cancer Surveillance Program (CSP) SEER registry. For both sites, cases were identified through rapid case ascertainment (RCA), which aims to identify cases within 3–6 months after diagnosis.

##### *Case sampling*.

We sampled from all eligible NHW 45–49 years of age due to budgetary constraints. Given that there is a paucity of studies among NHB women, the youngest women (< 45 years of age), and women diagnosed with estrogen receptor-negative tumors, we retained all NHB women diagnosed 20–49 years of age, all NHW women 20–44 years of age, and among NHW women aged 45–49 years, oversampled women with estrogen receptor-negative tumors. Thus, all eligible NHB cases 20–49 years of age and NHW cases 20–44 years of age were included, and a sample of NHW cases aged 45–49 years (n = 829 of 2,527 Detroit; n = 883 of 2,782 LA), sampled as follows: between 09/01/2010 and 08/31/2012 30.5% of all NHW 45–49 year old cases; between 08/31/2012 and 08/31/2015 84.5% of ER- cases and 40.8% of ER + tumors.

##### *Case screener interview*.

All sampled cases were screened to determine final eligibility status. Cases not successfully screened by a study site team were checked against the updated SEER Registry to determine eligibility status. Cases initially sampled were considered ineligible for the following reasons: not U.S.-born (n = 373), self-identified as neither White nor Black (n = 153), self-identified as Hispanic (n = 151), previous cancer diagnosis (n = 117), resided outside of the study areas at reference date (see definition of reference date in Table 1; n = 50), tumor had ineligible histology (n = 44), did not speak English (n = 29), updated age or reference date was out-of-range (n = 17), physically or mentally unable to complete the interview (n = 14), or institutionalized at reference date (n = 7). Two percent of cases were ineligible for screening for one or more of these reasons. In Detroit, a letter was sent to each eligible case’s physician before cases were contacted; if the physician did not respond within three weeks the case could be contacted, except for a few Detroit hospitals that required active physician approval.

#### Controls

YWHHS investigators and the Westat team developed the area-based control sampling strategy and Westat developed the statistical sampling methodology [48, 49]. Westat also oversaw control identification and recruitment, household rostering, screener interviews, and initiated control recruitment efforts. Once potentially eligible controls were identified, their contact information was provided to the YWHHS Coordinating Center to be entered into the study tracking database for recruitment.

##### *Control sampling*.

A three-stage area probability sample was conducted to provide coverage of metropolitan Detroit and LA County from which YWHHS case participants were identified (see Supplemental Materials). The first stage of sample selection was that of PSUs (Primary Sampling Units) consisting of one or more Census blocks as identified in the U.S. Census conducted in 2010. Within sampled PSUs, the second stage was the sampling of approximately 24,000 + addresses from listings based on addresses served by the U.S. Postal Service. Households within occupied sampled addresses were rostered to identify members who were potential controls for the study. The third stage of sample selection involved randomly selecting women from among those potentially eligible. The sampling rates employed were designed to obtain a set of controls that were frequency matched to the expected case distribution within study site by race (NHB/NHW) and 5-year age intervals.

##### *Control household roster*.

A total of 24,612 households were sampled (Table 2) and 21,668 were determined eligible for roster. An introductory letter, brief roster, and a $2 bill were mailed to all sampled residential addresses. The same follow-up household contact recruitment protocol was then used as the National Health and Nutrition Examination Survey [50]. A total of 18,612 household were rostered. The roster asked the initials/name, age, and race/ethnicity of all adult women 20–50 years old in the household (see Supplementary Materials for additional details).

**Table 2:** Overall ascertainment numbers by race and site, Young Women’s Health History Study

	Detroit				LA					Both Sites				
	NHB	NHW	Other	Total	NHB		NHW	Other	Total	NHB		NHW	Other	Total
	N	N	N	N	N		N	N	N	N		N	N	N
**Cases**														
All potentially eligible cases identified by SEER^a^	738	1,721	68	2,527	589		2,040	153	2,782	1,327		3,761	221	5,309
Sampled from potentially eligible cases	738	1,318	61	2,117	589		1,428	135	2,152	1,327		2,746	196	4,269
Not screened or incomplete screener	311	665	14	990	163		378	18	559	474		1,043	32	1,549
SEER Registry Determined Ineligible^b^	11	55	14	80	22		116	18	156	33		171	32	236
Hospital/physician refusal or active physician approval not received^c^	34	48	0	82	–		–	–	–	34		48	0	82
Participant not reached	94	407	0	501	123		232	0	355	217		639	0	856
Incomplete screener (eligible/potentially eligible)	172	155	0	327	18		30	0	48	190		185	0	375
Completed screener	427	653	47	1,127	426		1,050	117	1,593	853		1,703	164	2,720
Eligible	379	564	0	943	364		706	0	1,070	743		1,270	0	2,013
Ineligible	48	89	47	184	62		344	117	523	110		433	164	707
Eligible/potentially eligible (screened & not screened)	679	1,174	0	1,853	505		968	0	1,473	1,184		2,142	0	3,326
Not interviewed	296	638	0	934	206		374	0	580	502		1,012	0	1,514
Not screened—hospital/physician refusal or active physician approval not received^c^	34	48	0	82	–		–	–	–	34		48	0	82
Died before interview	26	34	0	60	40		24	0	64	66		58	0	124
Too ill to conduct interview	4	4	0	8	6		9	0	15	10		13	0	23
Participant refused	122	225	0	347	84		168	0	252	206		393	0	599
Proxy said participant refused	4	8	0	12	3		9	0	12	7		17	0	24
Moved away from study area	6	15	0	21	11		38	0	49	17		53	0	70
Unavailable for interview	100	304	0	404	62		126	0	188	162		430	0	592
Eligible and interviewed	383	536	0	919	299		594	0	893	682		1,130	0	1,812
**Controls**														
Households sampled	–	–	–	9,994	–		–	–	14,618	–		–	–	24,612
Total households ineligible for roster	–	–	–	1,418	–		–	–	1,526	–		–	–	2,944
Vacant households	–	–	–	1,284	–		–	–	709	–		–	–	1,993
Non-dwelling unit, e.g., institutional quarters	–	–	–	127	–		–	–	278	–		–	–	405
Excluded 60% of potential Hispanic households^d^	–	–	–	–	–		–	–	485	–		–	–	485
Language barrier	–	–	–	7	–		–	–	54	–		–	–	61
Total households eligible for roster	–	–	–	8,576	–		–	–	13,092	–		–	–	21,668
Households with no response^e^	–	–	–	774	–		–	–	2,282	–		–	–	3,056
Locked buildings/gated communities	–	–	–	91	–		–	–	1,197	–		–	–	1,288
Households rostered	–	–	–	7,802	–		–	–	10,810	–		–	–	18,612
By mail	–	–	–	2,802	–		–	–	3,333	–		–	–	6,135
In person^f^	–	–	–	5,000	–		–	–	7,427	–		–	–	12,477
Households with participants sampled for screening^g^	–	–	–	1,709	–		–	–	1,512	–		–	–	3,221
Households not screened	–	–	–	310	–		–	–	292	–		–	–	602
Participants potentially eligible & sampled^h,i^	643	1,038	73	1,761	624		897	125	1,653	1,929		1,261	198	3,414
Potentially eligible participants—incomplete screener^i,j^	98	251	7	362	127		190	13	336	222		441	20	698
Completed potential participant screener^i,k^	545	787	66	1,399	497		707	112	1,317	1,039		1,488	178	2,716
Ineligible participants	72	150	66	288	89		238	112	440	161		388	178	728
Eligible or potentially eligible participants	473	638	0	1,111	408		469	0	877	881		1,107	0	1,988
Didn’t agree to be contacted	12	18	0	30	10		15	0	25	22		33	0	55
Eligible	12	17	0	29	10		15	0	25	22		32	0	54
Unknown eligibility status	0	1	0	1	0		0	0	0	0		1	0	1
Agreed to be contacted	461	620	0	1,081	398		454	0	852	859		1,074	0	1,933
Not interviewed	109	255	0	364	85		103	0	188	194		358	0	552
Found to be ineligible	1	3	0	4	5		3	0	8	6		6	0	12
Died before interview	0	2	0	2	0		0	0	0	0		2	0	2
Too ill to conduct interview	0	1	0	1	0		1	0	1	0		2	0	2
Participant refused	51	147	0	198	48		67	0	115	99		214	0	313
Proxy said participant refused	2	4	0	6	3		5	0	8	5		9	0	14
Moved away from study area	8	10	0	18	3		9	0	12	11		19	0	30
Unavailable for interview^l^	47	88	0	135	26		18	0	44	73		106	0	179
Could not be located	1	2	0	3	1		2	0	3	3		3	0	6
Eligible & interviewed^m^	352	365	0	717	313		351	0	664	665		716	0	1,381

^a^Excludes NHB and NHW cases < 45 years of age identified by the SEER Registry post the study recruitment period (n = 146; DT: n = 74; LA: n = 72)

^b^Sampled/potentially eligible cases who did not complete a telephone screener and were determined to be ineligible based on SEER information

^c^Active physician approval required by specific hospitals among a subset of case participants in Detroit

^d^For efficiency, 60% of households identified by the Westat address list vendor as likely to include at least one “Hispanic” adult were randomly excluded. Information from the other 40% was used to impute adjusted sampling values

^e^Non-response households include those that refused, that were not reached after maximal contacts, that were locked buildings staff were unable to enter, or where language barriers existed

^f^7% and 18% of in-person rosters were completed by neighbors in Detroit and Los Angeles, respectively

^g^Households containing more than one potentially eligible and sampled woman (Detroit: 49; LA: 121)

^h^Of sampled potentially eligible participants, five lacked “race” values, 2 reported not knowing their self-selected “race,” and seven refused to report a “race” value

^i^If participants lacked a self-reported “race” value at the screener level, their reported “race” value from the household roster was used instead

^j^Of potentially eligible participants who did not complete a screener, 4 were missing “race” values, 1 reported not knowing their self-selected “race,” and 6 refused to report a “race” value

^k^Of participants who completed a screener, 1 reported not knowing their self-selected “race” and 1 refused to report a “race” value

^l^Includes participants lost (n = 6) or unable to contact to schedule an interview (n = 171)

^m^Households containing more than one eligible and interviewed participant (Detroit: 20; LA: 43)

##### *Control screener interview*.

An in-person screener interview was conducted to determine the final eligibility of potentially eligible women identified and sampled from the household roster. Those who completed the screener received $5. Respondents willing to participate or interested in learning more were asked to provide their contact information for a study site (WSU/USC) interviewer to contact them.

#### Data collection

##### *In-home case–control interview recruitment*.

An introductory letter and study brochure were sent to all sampled case and control women. After sending the introductory letter, study staff (WSU/USC) telephoned women to determine (cases) or confirm (controls) eligibility, answer questions, and identify a location and time for an in-person interview. Women not reached by phone were sent follow-up letters and reminder postcards, and, in some cases, in-person visits. Women who declined to participate were asked to complete a brief questionnaire about demographic characteristics to characterize non-respondents.

##### *In-person interview scheduling and informed consent*.

Study participants were interviewed at their selected location. Prior to interview, participants were mailed a confirmation letter and their interviewer’s business card with a photograph. Before the interview, the participant was asked to read and sign a consent form that described the study and participant rights and safeguards; it also requested permission to conduct the interview and each component of the study. Women were informed they could refuse any questions and terminate the interview at any time. Women who had a mammogram were asked to complete a separate consent form that requested permission to obtain information from her healthcare provider about her last mammogram before reference date. Additionally, case participants were asked to provide consent to obtain tumor tissue sampled at the time of diagnosis or thereafter. A thank you gift of $75, which was later increased to $100, was provided for the main interview.

##### *Main questionnaire*.

The YWHHS questionnaire captured information about energy balance factors (e.g., childhood and adult diet, physical activity, and adult body size), factors known to affect life-course energy balance (e.g., food security, sleep patterns, built environment), known risk factors for BC (e.g., reproductive and family history), as well as race/ethnicity and life-course socioeconomic indicators. Collected information related to race/ethnicity includes self-reported race and Hispanic ethnicity, as well as the race/ethnicity others typically ascribe to the participant. We also asked about early life discrimination, experiences of every-day discrimination and the source of discrimination. Life-course socioeconomic indicators include residential history, household percent poverty (HPP), educational attainment, and occupational status [51, 52]. HPP was calculated using household net income adjusted for household size. Other factors associated with social context collected include life-course experiences of adversity (including childhood experiences), financial status and use of governmental subsidies, food insecurity, occupational status, and health insurance status. Other information on factors potentially associated with BC risk include prenatal exposures, medical history, non-steroidal anti-inflammatory medication use, contraceptive use, hormone medication use, fertility history, and life-course personal and secondhand tobacco exposure, as well as alcohol use. Study questions were developed based on existing questionnaires [53–57].

Multiple tools were used throughout the questionnaire to assist participants with recall, including a life history calendar of key life events [58], showcards, which also provided a non-verbal method of responding to sensitive questions, and a photobook of oral contraceptive, hormone, and thyroid medications [58].

##### *Additional components of the in-person interview: anthropometric assessment*.

Height, weight, waist circumference, and body composition (assessed by Tanita bioelectrical impedance analysis (BIA)) were measured. *Diet.* A modified version of the full 100-item Block Food Frequency Questionnaire (FFQ) was developed by NutritionQuest (Berkeley, CA) with the study PI (Velie) to capture total diet and foods suspected to be associated with BC risk (e.g., cruciferous vegetables) in the 12 months prior to reference date. The FFQ was administered on paper or verbally during the interview; those who did not complete it at the interview returned it via mail or at the phlebotomy visit. Childhood diet was assessed with a food list. *Childhood photographs*. Participants provided photos from “head to toe” at ages 6, 9, 12, 15, and 18 years to validate recalled relative body size (assessed by somatotype); photos were scanned and de-identified by digitally masking the participant’s eyes/face, if requested. *Built environment survey*. Interviewers conducted a survey of neighborhood characteristics, primarily at the time of the interview [59, 60]. Surveys not completed by the end of study recruitment (6.5%) were conducted remotely via Google Maps Street View using photos collected at the date closest to the interview date [61]. *Primary caregiver survey* Participants were asked to mail their primary childhood caregiver a brief survey. Caregivers were given $10. The survey included respondent’s demographics, biologic mother’s pregnancy with the participant, and the study participant’s childhood body size, physical activity, and SEP.

#### Biospecimen collection

##### *Blood*.

All study participants were asked to provide a blood sample. Samples were collected by a phlebotomist, generally at the second visit (96%, 4% at first visit). Phlebotomists attempted to obtain 30 mL (approximately 2 tablespoons) collected in four 10-mL vacutainers: two with no additive and two with EDTA. For cases, our protocol indicated samples should not be collected until at least two months after last treatment (average days post treatment = 376 days; 95% CI 353.9, 398.6). Participants who provided blood samples were originally given a $20 thank you gift, which was later increased to $25. Samples were processed at the MSU Cytogenics laboratory and MCW Tissue Bank.

##### *Blood Questionnaire*.

Phlebotomists administered a questionnaire to each participant at the time of blood draw. Questions addressed recent medication use; medical history; menstrual, pregnancy, and lactation status; and recent food, beverage, alcohol, and tobacco consumption.

##### *Menstrual calendar*.

During the main interview, if a participant reported menstruating within the past year and if they consented to have their blood drawn, they were asked to complete a menstrual calendar that indicated each day they experienced menstrual bleeding until the date their blood was drawn. If participants had not completed this calendar at the time of blood draw, the phlebotomist completed it with the participant for the preceding two months.

##### *Menstrual postcard*.

At the end of the blood draw, menstruating participants were given a pre-addressed stamped postcard, and asked to record the date of the first day of their next menstrual cycle and mail it; this information was used to determine the participant’s menstrual phase at the time her blood was drawn.

##### *Saliva*.

Participants unwilling or unable to provide a blood sample were asked to provide a saliva sample with the Oragene OG-500 DNA kit. Saliva samples were collected immediately after administration of the main questionnaire, by the phlebotomist at the second visit, or mailed to the participant after the first visit and returned by mail.

##### *Tumor SEER Information*.

Tumors were characterized by ER, PR, and HER2 molecular subtypes, and histological grade to differentiate luminal A and luminal B tumors using data from SEER registries [11]. SEER reports also included ICD-O codes, tumor size, laterality, lymph node involvement, and initial treatment and surgical history.

##### *Tumor Tissue*.

To evaluate other tumor characteristics, e.g., Ki-67 status [11], tumor tissue from consenting cases was requested from hospitals or clinics where they were stored; when possible, tumor samples were taken before treatment. When adequate tissue was provided, tumor microarrays (TMAs) were created.

##### *Biospecimen storage.*

All blood, saliva, and tumor tissue biospecimens are stored at the MCW Tissue Bank as part of the YWHHS Biorepository. Separate biomarker studies will be conducted with all collected biospecimens.

#### Interviewer Training and Quality Control Measures

##### *Control recruitment interviewer training*.

Control field interviewers were employees of Westat. Interviewers from both study sites were trained together to synchronize data collection. Once they demonstrated adherence to all protocols they were certified for data collection.

##### *Study site interviewer and phlebotomist training*.

Training was conducted by the YWHHS Coordinating Center to synchronize data collection. All field staff completed appropriate IRB-mandated training and field safety training and were certified by the YWHHS Coordinating Center once they demonstrated adherence to all protocols and competence in a complete study interview.

##### *Main interview and phlebotomy quality control*.

Interviews and phlebotomy visits of consenting participants were audio recorded for quality control. The first five recorded interviews completed by each interviewer and additional interviews as needed based on performance (4.8% in Detroit; 2.6% in LA of completed interviews) were reviewed by a trained evaluator. The evaluator documented discrepancies in recorded responses, deviations from protocol, and appropriate probing, and provided detailed feedback to each interviewer.

### Study response and cooperation rate calculations

Response and cooperation rates were calculated using imputation methods in accordance with the American Association for Public Opinion (AAPOR) guidelines [62] (see Supplemental Tables 1 and 2).

### Sample weights

Sample weights were created for both cases and controls to account for sampling design and non-response. Weights reflect probabilities of selection and adjustments for non-response. Adjustments for non-response were done at the screener and main interview levels. To achieve the frequency matching of controls to cases, a weighted distribution of cases for each study site was established across cells of age and race. The sample weights of controls were then post-stratified to the weighted totals within each of these cells [63]. Additionally, replicate weights were created to develop estimates of variability, including standard errors. Demographic characteristics were obtained for 86% of sampled controls (complete roster information), and 100% of sampled case participants (age, race, site, county, ER status) to inform non-response weights. Replicate weights were created for case–control analyses and case-only analyses. A second set of weights was created for control-only analyses, to weight controls to the source population. Replicate weights were also created for blood sample analyses.

### Statistical analyses

Primary analyses are conducted using survey weighted multiple logistic regression to account for study design and potential confounding. Where appropriate, potential effect modification by study site, race and/or socioeconomic position are being evaluated. For some analyses, structural equation modeling (SEM) with latent variables is being conducted to evaluate exposures over the life-course [64]. Additionally, for some analyses we are using survey weighted polytomous logistic regression to assess heterogeneity in risk by tumor subtypes.

## Operational results

### Case participation

A total of 5,309 potentially eligible women were identified through the Detroit (n = 2,527) and LA (n = 2,782) SEER registries (Table 2). Of these, 80% were sampled (*see Case Sampling*), and 3,326 were determined to be eligible or potentially eligible (Table 2). Among sampled cases, 124 women died before they could be interviewed and 82 could not be contacted because physician or hospital permission was not obtained. Other reasons for non-interview included: 177 could not be located, 70 moved away from the study area, 23 were too ill, and 415 did not respond after maximum contact attempts. Of the 3,326 sampled and potentially eligible participants, study staff had the opportunity to recruit 2,435 participants. Of these, 623 declined to participate, and 1,812 women were interviewed (ER + n = 1,310; ER- n = 437). The overall cooperation rate was 74.4% (Detroit: 71.9%, LA: 77.2%) and response rate was 59.8% (Detroit: 53.1%, LA: 66.4%) (Supplemental Table 1). Response rates were higher for NHB women (60.2%) than NHW women (59.8%), and for LA (66.4%) than Detroit (53.1%) (Supplemental Table 1), but did not vary significantly by age (Supplemental Table 2).

### Control participation

A total of 24,612 households were sampled in Detroit (n = 9,994) and LA (n = 14,618) (Table 2). Of these, 21,668 were eligible or potentially eligible and 18,612 households completed a roster (86% response rate) (Supplemental Table 1). Households not rostered because they were in an inaccessible gated community included in LA 9% and Detroit 1% of potentially eligible households. Of households that completed rosters, 3,414 participants were sampled and 2,720 completed screeners (88% response rate, Supplemental Table 1). Reasons that screeners were not obtained were the following: resided outside the study area (n = 24), was too ill (n = 2), was not reached after maximum attempts (n = 132), or sampled in error (n = 9). Of the 3,247 participants sampled for screening that interviewers had the opportunity to screen, 83.6% were screened. Of these, 1,988 were eligible or potentially eligible and 97.2% agreed to be contacted by study site staff. Thus, Westat provided control participant information for 1,933 women. Of these, study site staff had no opportunity to interview 223 women for the following reasons: 12 were ineligible, 2 died before interview, 6 could not be located, 30 moved away from the study area, 2 were too ill, and 171 were not reached after the maximum number attempts. Thus, 1,708 participants were confirmed to be eligible and agreed to be contacted by the study site staff. Of these, 327 women refused to participate in the study (4% via proxy) and 1,381 completed the main interview (Table 2). Accounting for the household roster cooperation rate (94%), screener cooperation rate (84%), and study site recruitment cooperation rate (81%), the overall study cooperation rate was 65% (Supplemental Table 1). Similarly, taking into account the household roster response rate (86%), the participant screener response rate (88%), Westat agreed to be contacted response rate (98%), and the study site recruitment response rate (72%) led to an overall control response rate of 53% (supplemental Table 1). Response rates were higher for NHB women (57.9%) compared to NHW women (48.3%), and for LA (58.5%) compared to Detroit (49.3%) (Supplemental Table 1) but did not vary significantly by age (Supplemental Table 2).

### Main interview

#### Location of completed interviews

A total of 73.2% and 80.8% of interviews were conducted in-home, 3.4% and 3.0% were conducted at a study site office, and 23.5% and 16.2% were conducted at other locations (e.g., a coffee shop, local library, or healthcare provider’s office) for cases and controls, respectively. Distributions of interview locations were similar across study sites.

#### Interview timing

The median period between reference date and interview date was 153 days for controls and 378 days for cases (Supplemental Table 3).

**Table 3:** Weighted demographic characteristics of interviewed participants by site and case–control status, Young Women’s Health History Study (N = 3,193)

	Detroit				Los Angeles				Total			
	Case		Control		Case		Control		Case		Control	
	N	W Percent^a^	N	W Percent^a^	N	W Percent^a^	N	W Percent^a^	N	W Percent^a^	N	W Percent^a^
Total	919	100.0	717	100.0	893	100.0	664	100.0	1812	100.0	1381	100.0
Age at reference date												
20–29 years	23	2.3	94	2.2	45	3.6	152	3.6	68	2.9	246	2.9
30–34 years	63	5.7	99	5.6	77	6.5	132	6.4	140	6.0	231	6.0
35–39 years	164	13.8	123	13.8	143	12.6	128	12.7	307	13.2	251	13.3
40–44 years	337	30.6	201	31.3	337	29.4	110	29.4	674	30.0	311	30.4
45–49 years^b^	332	47.7	200	47.0	292	47.9	142	47.9	623	47.8	342	47.4
Race												
Non-Hispanic Black	383	32.5	352	32.5	299	27.6	313	27.6	682	30.2	665	30.2
Non-Hispanic White	536	67.5	365	67.5	594	72.4	351	72.4	1130	69.8	716	69.8
Household poverty												
< 150 percent	246	24.5	275	30.7	122	13.0	194	20.3	368	19.2	469	25.9
150 to < 300 percent	258	27.7	196	26.4	211	21.8	168	24.2	469	25.0	364	25.4
≥ 300 percent	371	43.5	220	39.5	531	61.8	285	54.0	902	52.0	505	46.3
Refused/don’t know/missing	44	4.3	26	3.3	29	3.4	17	1.5	73	3.8	43	2.4
Household poverty by race/ethnicity												
Non-Hispanic Black												
< 150 percent	168	43.1	200	54.2	73	24.4	159	42.3	241	35.1	359	49.1
150 to < 300%	99	25.7	86	25.8	97	32.9	70	25.3	196	28.8	156	25.6
≥ 300 percent	94	25.4	47	13.9	118	39.2	73	30.8	212	31.3	120	21.1
Non-Hispanic White												
< 150 percent	78	15.6	75	19.5	49	8.7	35	12.0	127	12.3	110	15.8
150 to < 300 percent	159	28.6	110	26.7	114	17.7	98	23.8	273	23.3	208	25.3
≥ 300 percent	277	52.2	173	51.9	413	70.4	212	62.8	690	61.0	385	57.2
Refused/Don’t Know/Missing	44	4.3	26	3.3	29	3.4	17	1.5	73	3.8	43	2.4
Employment (outside the home)												
None	168	17.2	189	25.4	162	18.7	152	26.5	330	17.9	341	25.9
Part-time (< 35 h a week)	144	16.8	101	15.6	134	15.8	106	18.4	278	16.3	207	16.9
Full-time (≥ 35 h a week)	568	62.8	368	54.4	549	60.4	338	50.4	1,117	61.3	706	52.5
Full-time student	30	2.9	56	4.2	33	3.6	67	4.4	63	3.2	123	4.3
Refused/don’t know/missing	9	1.0	3	0.3	15	1.5	1	0.4	24	1.3	4	0.3
Educational attainment												
Less than high school diploma	195	20.0	185	22.9	83	9.2	106	11.9	278	14.9	291	17.8
High school graduate or GED	353	36.8	300	40.0	294	32.2	261	41.6	647	34.7	561	40.7
Vocational school, associate’s degree, or some college	204	24.1	144	22.1	313	34.5	210	29.2	517	28.9	354	35.4
Bachelor’s degree or higher	167	19.1	88	15.0	200	23.9	87	17.3	367	21.3	175	16.1
Refused/don’t know/missing	0	0	0	0	3	0.2	0	0	3	0.1	0	0
Primary caregiver’s educational attainment												
Less than high school diploma	193	19.7	147	20.5	93	10.4	78	12.4	286	15.4	225	16.7
High school graduate or GED	356	41.5	268	38.7	263	32.8	191	30.0	619	37.5	459	34.6
Vocational school, associate’s degree, or some college	211	21.5	192	25.2	310	32.9	242	35.8	521	26.8	434	30.1
Bachelor’s degree or higher	107	12.4	72	10.8	204	21.3	139	18.9	311	16.5	211	14.6
Refused/don’t know/missing	52	5.0	38	4.8	22	2.5	14	2.9	74	3.7	52	3.9
Age at first birth												
Nulliparous	171	19.1	138	16.1	326	36.1	265	29.2	497	27.1	403	22.2
First birth age < 20 years	190	17.9	194	22.8	101	10.4	112	17.8	291	14.4	306	20.5
First birth age 20–29 years	385	42.4	278	41.5	234	25.5	194	29.6	619	34.5	472	35.9
First birth age ≥ 30 years	173	20.5	107	19.7	231	27.8	93	23.4	404	23.9	200	21.4
Missing	0	0	0	0	1	0.1	0	0	1	0.1	0	0
Birth cohort												
Born 1961–1969	459	59.2	287	60.3	428	59.9	173	56.1	887	59.5	460	58.4
Born 1970–1979	407	35.8	274	33.9	372	32.5	246	35.8	779	34.3	520	34.8
Born 1980–1989	51	4.8	130	5.1	87	7.3	197	7.6	138	6.0	327	6.3
Born 1990–1995	2	0.1	26	0.7	6	0.4	48	0.5	8	0.2	74	0.6

^a^Weighted percentages incorporate post-stratification sample weights

^b^Among 45–49 year old Non-Hispanic White women diagnosed with invasive BC, 48.7% of Detroit and 35.3% of LA cases were sampled; cases identified post the study recruitment (N = 258 (n = 66 Black; n = 192 White)) were considered not sampled; all other case subgroups were sampled at 100%

#### Length of main questionnaire

The questionnaire included 639 questions (excluding probing questions and repeat questions about exposures over the life-course). The median administration time of the questionnaire was 130 and 120 min for cases and controls, respectively (Supplemental Table 4). The median duration of the measured anthropometry section was 11 min for both cases and controls (Supplemental Table 4). Interview time for study participants was longer for NHB women (141 min) compared to NHW women (119 min) and for poorer women (HHP < 150; 132 min) compared to wealthier women (HHP ≥ 300; 120 min).

**Table 4:** Completion rates of study materials by case–control status and race, Young Women’s Health History Study

	Cases			Controls		
	Non-Hispanic Black	Non-Hispanic White	Total	Non-Hispanic Black	Non-Hispanic White	Total
	Percent	Percent	Percent	Percent	Percent	Percent
**Study material**						
Main interview (all eligible) (N):	(682)	(1130)	(1812)	(665)	(716)	(1381)
Completed all sections	98	99	99	99	99	99
Life history calendar	99	98	98	98	100	99
Anthropometry measurements						
Height, weight, waist/hip circumference	96	94	95	98	94	96
Bioelectric impedance assay^a^	81	88	85	82	87	85
Photographs of body size	18	55	41	19	52	36
Food Frequency Questionnaire	70	84	79	57	79	69
Neighborhood notes	88	91	90	92	96	94
Main interview audio consent	96	99	98	98	98	98
Main interview audio (of consented)	53	84	72	52	83	68
Residential census block information						
12 Months before reference date	95	96	96	96	96	96
Age 12	82	86	85	82	86	84
Permission to obtain health department information about participant’s birth	90	94	92	89	94	92
Permission to contact in future	98	99	99	99	99	99
Blood/saliva:						
Blood sample/saliva kit for DNA analyses	79	87	84	78	83	81
Blood sample	70	77	75	74	75	75
Blood Questionnaire^b^	99	99	99	99	99	99
Menstrual calendar^c^	88	93	92	90	94	92
Given and returned menstrual postcard^d^	43	69	61	42	63	53
Breast tumor:						
Tumor ER/PR/HER2 status from SEER	95	97	96	–	–	–
Tumor tissue consent received	96	97	96	–	–	–
Tumor tissue available of consented ^e^	78	50	60	–	–	–
Tumor tissue collected of available (as of 10/1/2020) ^e^	47	68	58	–	–	–
Among women who had mammogram (N):	(606)	(1045)	(1651)	(285)	(357)	(642)
Permission to obtain last mammogram	98	99	99	94	95	95
Among gravid women (N):	(548)	(766)	(1314)	(506)	(472)	(978)
Permission to obtain health department information about participant’s pregnancies	90	96	94	87	95	91
Among participants with eligible caregivers^F^ (N):	(500)	(973)	(1473)	(513)	(633)	(1146)
Caregiver survey	48	70	63	37	68	54

^a^Percentages based on number of participants who were not pregnant at the time of interview, N = 1659 cases and N = 1256 controls

^b^Percentages based on participants who completed blood draws

^c^Percentages based on participants who completed blood draws and were pre- or perimenopausal at time of blood draw

^d^Percentages based on participants who completed blood draws and were given menstrual postcards

^e^Tumor percent available based on number of participants with tumor material considered available of those who consented. Tumor availability determined by Slide Retrieval Program in LA and Epidemiology Research Core in Detroit. Primary reasons tumor not available were that there was not enough tumor tissue available for analysis and the hospital at which the specimen is stored does not allow researchers to take samples

^f^Percentages based on number of participants who completed interviews and who didn't report mother is “deceased” or “not in contact.”

#### Description of interviewed study population

Table 3 shows the weighted demographic characteristics of interviewed study participants. Cases were more likely to be wealthier than controls (52.0% vs. 46.3% HHP ≥ 300) and less likely to be unemployed (17.9% vs. 25.9%). Participants were similar across study sites, although both NHB and NHW women were more likely to be poor (HHP < 150%) in Detroit than LA. NHB women across both study sites were also significantly more likely to be poor (35.1% cases; 49.1% controls) compared to NHW women (12.3% cases; 15.8% controls) (Table 3).

#### Completion of study components

Response rates for all ancillary data collection efforts and for biospecimen collection are reported in Table 4. Nearly all participants completed the main interview (99%) and provided anthropometry measurements (95% of cases and 96% of controls). Most also provided blood samples (75% of cases and controls), or if blood was not provided, saliva (84% of cases and 81% of controls provided blood or saliva). In addition, 60% of women with BC who consented to allow us to retrieve tumor tissue had tissue available for analysis and thus far, of available participant tumor tissue, 58% has been retrieved (n = 660). Nearly all interviewed participants (97%) agreed to be contacted in the future.

## Discussion

We successfully conducted the YWHHS: a large population-based case–control epidemiologic study based on the eco-social theory of disease etiology [42] to identify potentially modifiable factors associated with young-onset BC overall and by molecular tumor subtypes, and to investigate racial and socioeconomic inequities in BC among NHB and NHW young women. For the extensive in-person interview (median time 120–130 min), we achieved a 60% response rate among cases and 53% response rate among controls, and the cooperation rate, among those we had the opportunity to interview was 74% among cases and 65% among controls. This was achieved through extensive follow-up efforts with the use of a centralized computer tracking system. Subsequently we achieved a high response rate to our request for blood (75%) or saliva samples when blood was not available (82%). With linkage to NCI SEER cancer registry data, we have valid information on the definition of a breast cancer case and detailed information on tumor subtype. With survey data linked to biospecimen information, we have collected comprehensive data to address this study’s research questions, as well as future studies of breast cancer. This is one the largest, population-based case–control studies of young-onset BC. Additionally, to our knowledge, this is the largest population-based case–control study of BC in young NHB women and the largest where extensive life-course individual-level socioeconomic measures were collected to evaluate racial and socioeconomic inequities in BC risk.

### Strengths

Strengths of this study include its exclusive focus on young women (aged < 50 years) incorporating information on tumor subtypes [9], and that it is designed to shed light on inequities in risk in young NHB compared to NHW women by life-course SEP. Other strengths include its population-based ascertainment of cases and controls and availability of created sample weights. The centralized YWHHS Coordinating Center synchronized data collection across study sites through conduct of all study interviewer and recruitment training and oversight, and through the study’s centralized tracking system. Other strengths include its in-depth assessment of social context, including residential history and current built environment. Additionally, biomarkers and both inherited genetic factors associated with BC and gene expression changes can be evaluated in this population-based study of young-onset BC—all of which are understudied.

### Limitations

Limitations of this study include potential residual recall bias for exposures that could not be validated. The study, however, used methods such as a life calendar, to minimize these issues [65]; life-course exposures were collected with recall aids, and YWHHS was able to validate recalled responses for key exposures, e.g., using measured adult and childhood photos to validate recalled anthropometry. The study sample size also limits our ability to examine young-onset BC risk by some rarer tumor subtypes and within some population subgroups for small effect sizes and more rare exposures; data from this study can be pooled with other studies to evaluate these questions. The timing of blood sample collection also prohibits examination of factors potentially affected by treatment or “case” status, though extensive information was collected to allow the study of these potential influences. Additionally, information on “race” is ultimately self-reported but was originally based on the SEER registry for cases. SEER registry reports of “race” and “Hispanic ethnicity,” however, are highly correlated with self-report [66, 67].

An additional limitation could be the study response rates; however, complete enumeration of cases in the SEER registry and 86% enumeration of sampled control households enabled us to incorporate non-response sample weights to mitigate this limitation. Declining response rates for national-level surveys, particularly telephone surveys, are well documented over the course of the survey period, and the challenges that caused this decline in rates also contributed to reduced response rates for YWHHS cases and controls [68]. Study response rates are, however, well within ranges reported in the literature [53, 69, 70], particularly for the data collection time period, participants’ ages, and the well-recognized challenges in enrolling disadvantaged populations [71, 72]. We found that women were more willing to participate when interviewers were similar in race and age (data not shown) [71, 73] and that response rates may have been lower among White women in Detroit due to interviewer-participant age incongruence. Recruitment and scheduling challenges included that women who were juggling childcare, work, other family responsibilities or challenging cancer treatment regimens often rescheduled interviews. To address these obstacles exclusive telephone schedulers were hired, targeted letters were mailed to address concerns regarding confidentiality and time constraints, in-person follow-up visits were attempted with controls in Detroit and cases and controls in LA, and the study incentive was increased.

### Future directions

Analyses using collected YWHHS data are in progress. Additional supplemental projects are possible, including pooling of data, particularly to study rarer tumor subtypes, studies to evaluate risk for other BC tumor subtypes, to study factors associated with mammograms and BC survival, to study biomarkers, e.g., gene expression, to integrate external data with data on geocoded life-course residential histories, and/or to evaluate intermediate biomarkers and BC risk. Results from YWHHS will expand our understanding of potentially modifiable factors associated with BC risk overall and by subtype and should identify sources of racial and socioeconomic inequities in young-onset BC.

## Supplementary Information

Below is the link to the electronic supplementary material.

Electronic supplementary material 1 (DOCX 48 kb)

## Data Availability

The datasets analyzed during the current study are not publicly available because main study findings are in process of being analyzed, but are available from the corresponding author on reasonable request.

## Abbreviations

AAPOR: American Association for Public Opinion
BC: Breast cancer
BIA: Bioelectrical impedance analysis
CAPI: Computer-assisted personal main interview
CARE: Contraceptive and Reproductive Endpoints
CPHS: California Committee for the Protection of Human Subjects
CRIC: Cancer Research Informatics Core
CSP: Cancer Surveillance Program
FFQ: Food Frequency Questionnaire
HR: Hormone receptor
IRB: Institutional review board
MCW: Medical College of Wisconsin
MDCSS: Metro Detroit Cancer Surveillance System
MSU: Michigan State University
NHB: Non-Hispanic Black
NHW: Non-Hispanic White
QC: Quality control
RCA: Rapid case ascertainment
RR: Response rate
SEER: Surveillance: Epidemiology and End Results
US: United States
USC: University of Southern California
UWM: University of Wisconsin—Milwaukee
W: Weighted
WSU: Wayne State University
YWHHS: Young Women Health History Study

## References

[1] Ward E et al (2019) Annual Report to the Nation on the Status of Cancer, 1999–2015, Featuring Cancer in Men and Women ages 20–49. J Natl Cancer Inst 10.1093/jnci/djz106 PMC6910179 31145458

[2] DeSantis C (2019). Breast cancer statistics, 2019. CA Cancer J Clin.

[3] Warner ET (2013). Reproductive factors and risk of premenopausal breast cancer by age at diagnosis: are there differences before and after age 40?. Breast Cancer Res Treat.

[4] White AJ (2015). Overall and central adiposity and breast cancer risk in the Sister Study. Cancer.

[5] Chollet-Hinton L (2016). Breast cancer biologic and etiologic heterogeneity by young age and menopausal status in the Carolina Breast Cancer Study: a case-control study. Breast Cancer Res.

[6] Assi H (2013). Epidemiology and prognosis of breast cancer in young women. J Thorac Dis.

[7] Nichols HB, Schoemaker MJ, Wright LB, McGowan C, Brook MN, McClain KM, Jones ME, Adami HO, Agnoli C, Baglietto L, Bernstein L (2017). The premenopausal breast cancer collaboration: a pooling project of studies participating in the National Cancer Institute Cohort Consortium. Cancer Epidemiol Prev Biomarks..

[8] Johnson KC, Glantz SA (2008). Evidence secondhand smoke causes breast cancer in 2005 stronger than for lung cancer in 1986. Prev Med.

[9] Barnard M, Boeke C, Tamimi R (2015). Established breast cancer risk factors and risk of intrinsic tumor subtypes. Biochem Biophys Acta.

[10] Perou CM (2000). Molecular portraits of human breast tumours. Nature.

[11] Goldhirsch A (2013). Personalizing the treatment of women with early breast cancer: highlights of the St Gallen International Expert Consensus on the Primary Therapy of Early Breast Cancer 2013. Ann Oncol.

[12] Balic M (2019). St. Gallen/Vienna 2019: a brief summary of the consensus discussion on the optimal primary breast cancer treatment. Breast Care.

[13] Shoemaker ML, White MC, Wu M, Weir HK, Romieu I (2018). Differences in breast cancer incidence among young women aged 20–49 years by stage and tumor characteristics, age, race, and ethnicity, 2004–2013. Breast Cancer Res Treat.

[14] Chen HL (2016). Effect of age on breast cancer patient prognoses: a population-based study using the SEER 18 Database. PLoS ONE.

[15] Eccles SA (2013). Critical research gaps and translational priorities for the successful prevention and treatment of breast cancer. Breast Cancer Res.

[16] Bray F (2018). Global Cancer Statistics 2018: GLOBCAN estimates of incidence and mortality worldwide for 36 cancers in 185 countries. Cancer.

[17] Henley A (2020). Annual Report to the Nation on the Status of Cancer, Part 1: National Cancer Statistics. Cancer.

[18] Singh GK, Jemal A (2017). Socioeconomic and racial/ethnic disparities in cancer mortality, incidence, and survival in the United States, 1950–2014: over six decades of changing patterns and widening inequalities. J Environ Public Health.

[19] SEER*Explorer Application: Breast Cancer Recent Trends in SEER Age-Adjusted Mortality Rates, 2000–2018 by Race/Ethnicity, Female, Ages <50, SEER, Editor. 2020.

[20] SEER*Explorer Application: Breast Cancer Recent Trends in SEER Age-Adjusted Incidence Rates, 2000–2017 by Race/Ethnicity, Female, Ages 15–39, All Stages, Delay-adjusted Rates, SEER, Editor. 2020.

[21] Qin B (2021). Neighborhood social environmental factors and breast cancer Subtypes among Black Women. Cancer Epidemiol Biomarkers Prev.

[22] Linnenbringer E (2020). Associations between breast cancer subtype and neighborhood socioeconomic and racial composition among Black and White women. Breast Cancer Res Treat.

[23] Taylor TR (2007). Racial discrimination and breast cancer incidence in US Black women: the Black Women's Health Study. Am J Epidemiol.

[24] Lu G (2018). The fluctuating incidence, improved survival of patients with breast cancer, and disparities by age, race, and socioeconomic status by decade, 1981–2010. Cancer Manag Res.

[25] Andaya AA (2012). Socioeconomic disparities and breast cancer hormone receptor status. Cancer Causes Control.

[26] Colditz GA, Bohlke K, Berkey CS (2014). Breast cancer risk accumulation starts early: prevention must also. Breast Cancer Res Treat.

[27] Kawai M (2014). Height, body mass index (BMI), BMI change, and the risk of estrogen receptor-positive, HER2-positive, and triple-negative breast cancer among women ages 20 to 44 years. Cancer.

[28] Ma H (2015). Reduced risk of breast cancer associated with recreational physical activity varies by HER2 status. Cancer Med.

[29] Premenopausal Breast Cancer Collaborative, G et al (2018) Association of Body Mass Index and age with subsequent breast cancer risk in premenopausal women. JAMA Oncol 4(11):e181771 10.1001/jamaoncol.2018.1771 PMC6248078 29931120

[30] Robinson WR (2014). Body size across the life course and risk of premenopausal and postmenopausal breast cancer in Black women, the Carolina Breast Cancer Study, 1993–2001. Cancer Causes Control.

[31] Xue F (2016). Body fatness throughout the life course and the incidence of premenopausal breast cancer. Int J Epidemiol.

[32] Millikan RC (2008). Epidemiology of basal-like breast cancer. Breast Cancer Res Treat.

[33] Chollet-Hinton L (2017). Biology and Etiology of Young-Onset Breast Cancers among Premenopausal African American Women: Results from the AMBER Consortium. Cancer Epidemiol Biomarkers Prev.

[34] Bandera EV (2015). Obesity, body fat distribution, and risk of breast cancer subtypes in African American women participating in the AMBER Consortium. Breast Cancer Res Treat.

[35] Krieger N (1994). Epidemiology and the web of causation: has anyone seen the spider?. Soc Sci Med.

[36] Krieger N (2020). Measures of racism, sexism, heterosexism, and gender binarism for health equity research: from structural injustice to embodied harm-an ecosocial analysis. Annu Rev Public Health.

[37] Omi M, Winant H (1994). Racial formation in the United States: from the 1960's to the 1990's.

[38] Jones C (2000). Levels of racism: a theoretic framework and a gardener's tale. Am J Public Health.

[39] Duster T (2005). MEDICINE: enhanced: race and reification in science. Science.

[40] Williams DR, Mohammed SA, Shields AE (2016). Understanding and effectively addressing breast cancer in African American women: Unpacking the social context. Cancer.

[41] Ford CL, Harawa NT (2010). A new conceptualization of ethnicity for social epidemiologic and health equity research. Soc Sci Med.

[42] Krieger, N., Ecosocial Theory of Disease Distribution: Embodying Societal & Ecologic Context, in Epidemiology and the People's Health. Theory and Context. 2013. p. 202–235.

[43] Williams DR, Mohammed SA (2009). Discrimination and racial disparities in health: evidence and needed research. J Behav Med.

[44] Linnenbringer E, Gehlert S, Geronimus AT (2017). Black-White disparities in breast cancer subtype: the intersection of socially patterned stress and genetic expression. AIMS Public Health.

[45] Jones CP (2002). Confronting institutionalized racism. Phylon.

[46] Jones CP (2001) Invited commentary: "race," racism, and the practice of epidemiology. Am J Epidemiol 154(4):299–304; discussion 305–6. 10.1093/aje/154.4.299 11495851

[47] Krieger N (2013). History, biology, and health inequities: emergent embodied phenotypes and the illustrative case of the breast cancer estrogen receptor. Am J Public Health.

[48] DiGaetano R, Waksberg J (2002). Commentary: trade-offs in the development of a sample design for case-control studies. Am J Epidemiol.

[49] Brogan DJ (2001). Comparison of telephone sampling and area sampling: response rates and within-household coverage. Am J Epidemiol.

[50] National Health And Nutrition Examination Survey III: Field Operations Manual. 1991.

[51] Mesenbourg T et al (2010) Census Summary File 1

[52] Jones CP (2008). Using "socially assigned race" to probe white advantages in health status. Ethn Dis.

[53] Marchbanks PA (2002). The NICHD Women's Contraceptive and Reproductive Experiences Study: methods and operational results. Ann Epidemiol.

[54] Gammon MD (2002). The Long Island Breast Cancer Study Project: description of a multi-institutional collaboration to identify environmental risk factors for breast cancer. Breast Cancer Res Treat.

[55] Brinton LA (1995). Oral contraceptives and breast cancer risk among younger women. J Natl Cancer Inst.

[56] Resnick M, Bearman P, Blum R (1997). Protecting adolescents from harm. Findings from the National Longitudinal Study on Adolescent Health. JAMA.

[57] Hamilton C, Strader L, Pratt J (2011). The PhenX Toolkit: get the most from your measures. Am J Epidemiol.

[58] Wingo P (1988). The evaluation of the data collection process for a multicenter, population-based, case-control design. Am J Epidemiol.

[59] Pebley NSA (2003). Neighborhood and family effects on children's health in Los Angeles.

[60] Weir SS Healthy environments partnership neighborhood observational checklist. University of Michigan, Ann Arobor, pp 1–12.

[61] Kelly C (2013). Using Google Street View to audit the built environment: inter-rater reliability results. Ann Behav Med.

[62] AAPOR (2016). Standard definitions: final dispositions of case codes and outcome rates for surveys.

[63] Li Y, Graubard B, Digaetano R (2011). Weighting methods for population-based case-control studies with complex sampling. J R Stat Soc Ser C.

[64] Bollen K (1989). Structural equations with latent variables.

[65] Mahabir S (2012). Challenges and opportunities in research on early-life events/exposures and cancer development later in life. Cancer Causes Control.

[66] Gomez SL, Glaser SL (2006). Misclassification of race/ethnicity in a population-based cancer registry (United States). Cancer Causes Control.

[67] Hamilton A (2009). Latinas and breast cancer outcomes: population-based sampling, ethnic identity, and acculturation assessment. Cancer Epidemiol Biomarkers Prev.

[68] Tourangeau R, Plewes (2013) Nonresponse in social science surveys: a research agenda. National Academy of Sciences, Washington, DC

[69] Xu M (2018). Response rates in case-control studies of cancer by era of fieldwork and by characteristics of study design. Ann Epidemiol.

[70] Palmer JR, Ambrosone CB, Olshan AF (2014). A collaborative study of the etiology of breast cancer subtypes in African American women: the AMBER consortium. Cancer Causes Control.

[71] Pinn V et al (2003) Outreach Notebook: For the inclusion, recruitment and retention of women and minority subjects in clinical research, U.S.D.o.H.a.H. Services, Editor. National Institutes of Health

[72] Bartlett DWR (2013). Recruitment and retention of African American and Hispanic girls and women in research. Public Health Nurs.

[73] Moorman PG (1999). Participation rates in a case-control study: the impact of age, race, and race of interviewer. Ann Epidemiol.

[74] Schwartz K (2013). Enhancement and validation of an Arab surname database. J Registry Manag.

[75] Williams DR (1997). Race and health: basic questions, emerging directions. Ann Epidemiol.

[76] Folsom R, otter F, Williams SRTI (1987) Notes on a composite size measure for self-weighting samples in multiple domains. American Statistical Association Meeting, pp 792–796

