# Supplementary material for: Theory, methods, and operational results of the Young Women’s Health History Study: a study of young-onset breast cancer incidence in Black and White women
Source: Cancer Causes Control. 2021 Jul 22;32(10):1129–48. doi: 10.1007/s10552-021-01461-x (PMC8416838; doi:10.1007/s10552-021-01461-x)
Supplement: Supplementary file 1 — Electronic supplementary material 1 (DOCX 48 kb) [file 10552_2021_1461_MOESM1_ESM.docx]

**SUPPLEMENTARY INFORMATION**

**Detailed Description of Control Sampling**

***Control sampling.*** A three-stage area probability sample was conducted to provide coverage of the Metropolitan Detroit (Oakland, Wayne, and Macomb County), MI and LA County, CA areas from which YWHHS case participants were identified. The first sampling stage created a frame of 1,000 primary sampling units (PSUs) (500 per study site) based on U.S. Census 2010 census blocks and U.S. postal addresses. PSUs were selected with probability proportionate to size; the size measure was developed to obtain about two study interviews per sample PSU [76]. Second, an address-based sample was selected from postal addresses associated with sampled PSUs using an equal probability sampling method. Sampled addresses were then screened using a household roster, and potentially eligible controls were sampled from potentially eligible participants. The sampling rate within each PSU could vary with the first-stage selection probability such that the combined selection probability would be equal across all housing units. For budgetary reasons, LA County census blocks in which a high proportion of households self-reported race as other than NHB or NHW were excluded from both the case and control sampling frame (a loss of an estimated 3-4% of NHW and 5-6% of NHB potentially eligible participants). Additionally, 60% of households identified by the Westat address list vendor as “Hispanic” were randomly excluded and information from the other 40% of these households was used to impute adjusted sampling values. The third sampling stage involved randomly selecting from all eligible controls, frequency-matched to cases within study site by race (NHB/NHW) and 5-year age intervals.

***Control household roster.*** ***Household roster recruitment efforts.*** First, an introductory letter, brief roster, and a $2 bill were mailed to all sampled residential addresses. A postcard followed by a second letter and the brief roster was sent to households that had not returned the roster after two weeks. If the household did not return their roster one month after it had first been mailed, a field interviewer attempted to roster the household in-person. Roster information was entered into an electronic questionnaire and a sampling algorithm run to select controls among potentially eligible participants. If in-person contact with a household was not made on the first attempt, the interviewer left a “Sorry I Missed You” card with a brief description of why they were there, the interviewer’s name, and Westat’s toll-free respondent phone number. Westat then used the same follow-up household contact recruitment protocol as the National Health and Nutrition Examination Survey [50].

Two challenges affected Westat control enrollment in LA County. One involved screening study participants who resided in gated communities (9% of potentially eligible households in LA). The Westat team, however, was able to successfully complete 83% of household rosters in LA and 91% of household rosters in Detroit; this information was used to create non-response control sample weights. Additionally, the proportion of NHB controls identified as ineligible due to non-NHB race/ethnicity based on household roster was higher than anticipated based on the LA County 2010 Census; our data suggested this was due to encountering a higher than anticipated Hispanic population. Westat addressed this limitation through a revision to our sampling methodology.

**Coding, editing, data management**

Screener questionnaire information was collected via an on-line Computer Assisted Personal Interview (CAPI). Main interview responses were collected via an off-line CAPI and uploaded into the study tracking system once secure network access was available. Participant age, site, case-control status, and date of diagnosis were pre-populated into the main CAPI interview after confirmation by interviewers during the screening interview. The CAPI enforced skip logic and implemented range or validity checks where appropriate. Data from the FFQ, Neighborhood Survey, Primary Caregiver’s Survey, Blood Questionnaire, Menstrual Calendar, and Menstrual Postcard were collected on paper forms that had barcodes and were returned to the study site field offices. Data were entered at the study sites and subsequently at the YWHHS Coordinating Center to check for errors and discrepancies. FFQs were collected on paper Scantron forms and processed by NutritionQuest.

**Supplemental Table 1. Interview response rates^a^ (RR) and cooperation rates^b^ (CR) by site, case-control status, and race, Young Women’s Health History Study**

|  | **Cooperation Rate**^b^ | | | **Response Rate**^a^ | | |
| --- | --- | --- | --- | --- | --- | --- |
|  | **Detroit** | **Los Angeles** | **Overall** | **Detroit** | **Los Angeles** | **Overall** |
|  | **Percent** | **Percent** | **Percent** | **Percent** | **Percent** | **Percent** |
| **Cases** |  |  |  |  |  |  |
| *Participant Screener* |  |  |  |  |  |  |
| Non-Hispanic Black | 61.6 | 75.1 | 67.7 | 57.9 | 72.3 | 64.3 |
| Non-Hispanic White | 53.7 | 80.0 | 67.4 | 49.5 | 73.5 | 62.0 |
| Total | 56.6 | 78.9 | 67.5 | 52.5 | 73.2 | 62.8 |
| *Study Site Recruitment* |  |  |  |  |  |  |
| Non-Hispanic Black | 75.2 | 77.5 | 76.2 | 59.4 | 59.2 | 59.3 |
| Non-Hispanic White | 99.8 | 78.0 | 87.0 | 47.6 | 61.4 | 54.0 |
| Total | 87.9 | 77.8 | 82.6 | 51.9 | 60.6 | 55.9 |
| *Overall* |  |  |  |  |  |  |
| Non-Hispanic Black | 75.3 | 77.5 | 76.2 | 58.6 | 62.2 | 60.2 |
| Non-Hispanic White | 69.7 | 77.0 | 73.4 | 50.1 | 68.4 | 59.8 |
| Total | 71.9 | 77.2 | 74.4 | 53.1 | 66.4 | 59.8 |
| **Controls** |  |  |  |  |  |  |
| *Household Roster* |  |  |  |  |  |  |
| Total | 96.2 | 95.2 | 95.6 | 91.0 | 82.6 | 85.9 |
| *Participant Screener* |  |  |  |  |  |  |
| Non-Hispanic Black | 90.4 | 82.2 | 86.5 | 87.3 | 90.2 | 88.7 |
| Non-Hispanic White | 82.5 | 80.3 | 81.6 | 80.6 | 93.9 | 86.4 |
| Total | 85.5 | 81.7 | 83.6 | 83.6 | 92.7 | 87.8 |
| *Agreed to be Contacted^c^* |  |  |  |  |  |  |
| Non-Hispanic Black | 98.7 | 97.3 | 97.5 | 97.5 | 97.5 | 97.5 |
| Non-Hispanic White | 96.1 | 97.2 | 97.0 | 97.2 | 96.8 | 97.0 |
| Total | 97.1 | 97.2 | 97.2 | 97.3 | 97.1 | 97.5 |
| *Study Site Recruitment* |  |  |  |  |  |  |
| Non-Hispanic Black | 85.6 | 86.0 | 86.6 | 76.5 | 79.6 | 78.0 |
| Non-Hispanic White | 71.7 | 82.2 | 76.2 | 59.2 | 77.8 | 67.0 |
| Total | 77.9 | 84.4 | 80.9 | 66.6 | 78.7 | 71.9 |
| *Overall* |  |  |  |  |  |  |
| Non-Hispanic Black | 74.5 | 67.3 | 71.6 | 59.3 | 57.4 | 57.9 |
| Non-Hispanic White | 56.9 | 62.9 | 59.4 | 42.1 | 58.9 | 48.3 |
| Total | 64.0 | 65.7 | 64.7 | 49.3 | 58.5 | 52.9 |

^a^Response rate was calculated using AAPOR RR4. RR4 = Interviewed/(Interviewed + (Refusal(Participant or Physician) + Non-Contact + Other(Too Ill, Could Not be Located, Moved away from Study Area, Died before Interview)) [62].

^b^Cooperation rate was calculated using AAPOR COOP3. RR4 = Interviewed/(Interviewed + Refusal(Participant or Physician)) [62].

**Supplemental Table 2. Interview response rates^a^ (RR) and cooperation rates^b^ (CR) by site, case-control status, race and age, Young Women’s Health History Study**

|  | **Cooperation Rate** | | | **Response Rate** | | |
| --- | --- | --- | --- | --- | --- | --- |
| **Age at reference date (years)** | **<40** | **40-44** | **45-49** | **<40** | **40-44** | **45-49** |
|  | **Percent** | **Percent** | **Percent** | **Percent** | **Percent** | **Percent** |
| **Cases** |  |  |  |  |  |  |
| **Los Angeles** |  |  |  |  |  |  |
| Non-Hispanic Black | 75.8 | 70.5 | 69.4 | 64.9 | 62.0 | 60.9 |
| Non-Hispanic White | 71.5 | 72.7 | 75.1 | 67.9 | 67.8 | 70.3 |
| **Detroit** |  |  |  |  |  |  |
| Non-Hispanic Black | 72.7 | 70.4 | 65.8 | 61.1 | 60.7 | 55.4 |
| Non-Hispanic White | 66.4 | 60.0 | 62.4 | 52.7 | 48.6 | 49.8 |
| **Overall** |  |  |  |  |  |  |
| Non-Hispanic Black | 74.0 | 70.5 | 67.5 | 62.6 | 61.2 | 57.9 |
| Non-Hispanic White | 69.2 | 66.4 | 67.6 | 60.3 | 57.6 | 57.4 |
| **Total** | 70.7 | 67.6 | 67.5 | 62.0 | 59.7 | 58.3 |
|  |  |  |  |  |  |  |
| **Controls** |  |  |  |  |  |  |
| **Los Angeles** |  |  |  |  |  |  |
| Non-Hispanic Black | 62.9 | 71.3 | 69.0 | 58.5 | 57.7 | 55.7 |
| Non-Hispanic White | 68.8 | 55.3 | 63.8 | 60.2 | 51.5 | 62.1 |
| **Detroit** |  |  |  |  |  |  |
| Non-Hispanic Black | 56.9 | 74.8 | 80.2 | 54.2 | 60.6 | 67.4 |
| Non-Hispanic White | 62.3 | 50.9 | 57.3 | 42.2 | 39.3 | 45.0 |
| **Overall** |  |  |  |  |  |  |
| Non-Hispanic Black | 72.9 | 74.4 | 70.3 | 56.3 | 58.1 | 61.4 |
| Non-Hispanic White | 63.2 | 55.1 | 61.7 | 50.0 | 42.1 | 50.2 |
| **Total** | 67.7 | 62.4 | 65.3 | 53.3 | 48.3 | 54.8 |

^a^Among 45-49 year old Non-Hispanic White women diagnosed with invasive BC, 48.7% of Detroit and 35.3% of LA cases were sampled; cases identified post recruitment (N=258 (n=66 Black; n=192 White) were considered not sampled; all other case subgroups were sampled at 100%.

**Supplemental Table 3. Time from reference date to interview date (days) by site, case-control status, and race, Young Women’s Health History Study**

|  | **Time from Reference to Interview Date**  **No. of Days** | | | |
| --- | --- | --- | --- | --- |
|  |  |  |  |  |
|  | **Mean (+/- SD)** | **25%** | **50%** | **75%** |
| **Cases** |  |  |  |  |
| **Los Angeles** |  |  |  |  |
| Non-Hispanic Black | 450 (235) | 252 | 383 | 615 |
| Non-Hispanic White | 469 (254) | 258 | 403 | 609 |
| **Detroit** |  |  |  |  |
| Non-Hispanic Black | 370 (262) | 174 | 300 | 482 |
| Non-Hispanic White | 468 (295) | 239 | 413 | 632 |
| **Overall** |  |  |  |  |
| Non-Hispanic Black | 405 (254) | 216 | 343 | 533 |
| Non-Hispanic White | 469 (274) | 250 | 405 | 622 |
| **Total** | 445 (268) | 235 | 378 | 588 |
|  |  |  |  |  |
| **Controls** |  |  |  |  |
| **Los Angeles** |  |  |  |  |
| Non-Hispanic Black | 193 (121) | 134 | 146 | 193 |
| Non-Hispanic White | 211 (155) | 141 | 156 | 197 |
| **Detroit** |  |  |  |  |
| Non-Hispanic Black | 215 (180) | 135 | 147 | 187 |
| Non-Hispanic White | 231 (178) | 143 | 164 | 226 |
| **Overall** |  |  |  |  |
| Non-Hispanic Black | 205 (155) | 135 | 147 | 188 |
| Non-Hispanic White | 221 (167) | 141 | 160 | 211 |
| **Total** | 213 (162) | 138 | 153 | 199 |

~~Note: N=3,193; n=0 with missing data; n=41 with SEER/YWHHS >2 month discrepancy regarding diagnosis date (these values are based on YWHHS reference date)~~

**Supplemental Table 4.** Duration of Main Questionnaire Interview (minutes) by Site, Case-Control Status, and Race, Young Women’s Health History Study Among Completed Interviews*

|  | **Duration of Main Questionnaire Interview**  **No. of Minutes** | | | |
| --- | --- | --- | --- | --- |
|  |  |  |  |  |
|  | **Mean (+/- SD)** | **25%** | **50%** | **75%** |
| **Cases** |  |  |  |  |
| **Los Angeles** |  |  |  |  |
| Non-Hispanic Black | 160 (30) | 140 | 158 | 180 |
| Non-Hispanic White | 131 (27) | 114 | 127 | 150 |
| **Detroit** |  |  |  |  |
| Non-Hispanic Black | 150 (38) | 120 | 145 | 180 |
| Non-Hispanic White | 116 (23) | 100 | 112 | 128 |
| **Overall** |  |  |  |  |
| Non-Hispanic Black | 154 (35) | 130 | 150 | 180 |
| Non-Hispanic White | 124 (26) | 105 | 120 | 137 |
| **Total** | 135 (33) | 111 | 130 | 152 |
|  |  |  |  |  |
| **Controls** |  |  |  |  |
| **Los Angeles** |  |  |  |  |
| Non-Hispanic Black | 141 (31) | 120 | 136 | 160 |
| Non-Hispanic White | 118 (23) | 105 | 120 | 129 |
| **Detroit** |  |  |  |  |
| Non-Hispanic Black | 135 (36) | 111 | 126 | 155 |
| Non-Hispanic White | 108 (24) | 92 | 106 | 121 |
| **Overall** |  |  |  |  |
| Non-Hispanic Black | 138 (34) | 115 | 132 | 158 |
| Non-Hispanic White | 113 (24) | 96 | 111 | 122 |
| **Total** | 125 (32) | 105 | 120 | 141 |

*N=3193 minus 37; n=7 with partially completed interviews; n=8 with data not uploaded and audio and life history calendar etc. used to reconstruct interview; 22 missing section A-Q interview time

**Supplemental Table 5.** Duration of Measured Anthropometry (minutes) by Site, Case-Control Status, and Race, Young Women’s Health History Study Among Completed Interviews*

|  | **Duration of Measured Anthropometry**  **No. of Minutes** | | | |
| --- | --- | --- | --- | --- |
|  |  |  |  |  |
|  | **Mean (+/- SD)** | **25%** | **50%** | **75%** |
| **Cases** |  |  |  |  |
| **Los Angeles** |  |  |  |  |
| Non-Hispanic Black | 13 (5) | 10 | 10 | 15 |
| Non-Hispanic White | 13 (5) | 11 | 14 | 15 |
| **Detroit** |  |  |  |  |
| Non-Hispanic Black | 17 (21) | 10 | 10 | 13 |
| Non-Hispanic White | 10 (3) | 9 | 10 | 11 |
| **Overall** |  |  |  |  |
| Non-Hispanic Black | 15 (16) | 10 | 10 | 15 |
| Non-Hispanic White | 11 (4) | 9 | 11 | 15 |
| **Total** | 13 (11) | 10 | 11 | 15 |
|  |  |  |  |  |
| **Controls** |  |  |  |  |
| **Los Angeles** |  |  |  |  |
| Non-Hispanic Black | 13 (5) | 10 | 12 | 15 |
| Non-Hispanic White | 13 (5) | 11 | 15 | 15 |
| **Detroit** |  |  |  |  |
| Non-Hispanic Black | 18 (20) | 10 | 10 | 15 |
| Non-Hispanic White | 11 (4) | 9 | 10 | 12 |
| **Overall** |  |  |  |  |
| Non-Hispanic Black | 16 (15) | 10 | 11 | 15 |
| Non-Hispanic White | 12 (4) | 10 | 12 | 15 |
| **Total** | 14 (11) | 10 | 11 | 15 |

*N=3193 minus 42; n=10 with partially completed interviews; n=8 with data not uploaded and audio and life history calendar etc. used to reconstruct interview; 24 missing section R interview time
